# Host-specific microbiome-rumination interactions shape methane-yield phenotypes in dairy cattle

**DOI:** 10.1128/msphere.00090-25

**Published:** 2025-04-25

**Authors:** Alejandro Castaneda, Nagaraju Indugu, Kathryn Lenker, Kapil Narayan, Sarah Rassler, Joseph Bender, Linda Baker, Ojas Purandare, David Chai, Xin Zhao, Dipti Pitta

**Affiliations:** 1 Department of Clinical Studies, School of Veterinary Medicine, University of Pennsylvania 70736 , Philadelphia, Pennsylvania, USA; 2 Department of Animal Science, Faculty of Agricultural and Environmental Sciences, McGill University 151165 , Sainte-Anne-de-Bellevue, Québec, Canada; University of Wisconsin-Madison, Madison, Wisconsin, USA

**Keywords:** rumination, eating, methane emissions, methane-yield phenotype, rumen bacteria

## Abstract

Enteric methane emissions (EMEs) negatively impact both the environment and livestock efficiency. Given the proposed link between CH_4_ yield and the rumination time (RT) phenotype, we hypothesize that this connection is mediated by the gut microbiome. This study investigated the RT-microbiome-EME connection using rumination-bolus, fecal, and rumen microbiomes as non-invasive proxies for identifying low-EME cows. High-RT cows ruminated 94 minutes longer per day (20%) and exhibited 26% lower EME than low-RT cows, confirming a strong RT-CH_4_-yield association. Microbial analysis revealed conserved methanogen diversity across the rumen, bolus, and fecal microbiomes, though functional differences were evident. High-RT cows had a greater abundance of *Methanosphaera stadtmanae*, suggesting an increased potential for methylotrophic methanogenesis, whereas low-RT cows exhibited higher *Methanobrevibacter* YE315 abundance, indicative of CO_2_-utilizing methanogenesis. Additionally, high-RT cows showed increased alternative hydrogen sinks, supported by upregulated genes encoding fumarate reductase, sulfate reductase, nitrate reductase, and ammonia-forming nitrite reductase, thereby reducing hydrogen availability for methanogenesis. Metabolically, high-RT cows had higher propionate concentrations and were enriched with rapid-fermenting bacteria (*Prevotella*, *Sharpea*, *Veillonellaceae*, and *Succinivibrionaceae*), whereas low-RT cows exhibited higher acetate concentrations with elevated acetate-producing pathways, reflecting differences in energy partitioning mechanisms. This study establishes RT as a microbiome-linked, non-invasive screening tool for identifying low-EME cows. The observed microbial and metabolic shifts in high-RT cows suggest that RT-based selection could enhance methane mitigation, rumen efficiency, and climate-smart livestock production. Leveraging RT-associated microbial profiles offers a scalable and cost-effective approach to reducing EME in cattle.

**IMPORTANCE:**

Methane emissions from livestock contribute to climate change and reduce animal efficiency. This study reveals that cows with longer rumination times (chewing cud for an extra 94 minutes daily) produce 26% less methane than cows with shorter rumination times. The gut microbiome plays a key role—low-methane cows host microbial communities that produce less methane while efficiently utilizing hydrogen for energy conservation in the rumen. By analyzing rumination sensor data and/or in combination with microbial profiles from rumen or fecal samples, farmers can non-invasively identify and select cows that naturally emit less methane. This scalable, cost-effective strategy offers a practical solution for reducing livestock’s environmental footprint while enhancing efficiency and advancing climate-smart agriculture.

## INTRODUCTION

Enteric methane emissions (EMEs) constitute a complex and long-unresolved problem that has a considerable negative impact on animal productivity and the environment. They represent an energy-inefficient process in which cattle lose between 2% and 12% of the gross energy consumed, decreasing their efficiency (1, 2). According to the “Global Methane Budget,” EME contributes 15.4% of the total CH_4_ emissions and 54% of agriculture’s CH_4_ emissions globally, where livestock is the main contributor (3). In the case of dairy cattle, they contribute 30% of agriculture’s greenhouse gas (GHG) globally (4). Moreover, since pre-industrial times, the concentration of CH_4_ in the atmosphere has increased by 162% (5), which, in synergy with its high potential to absorb the infrared radiation from the sun, is altering the planet’s temperature and exacerbating the effects of global warming (6–8). Hence, EME from dairy cattle entails adverse side effects on animal productivity and the environment that must be addressed.

Mitigating EME requires understanding multiple animal components, including the rumen microbiome. The rumen microbiome is diverse and morphs depending on diet, health status, and animal phenotype (9–11). With the rise of next-generation sequencing tools, studies to understand the link between the rumen microbiome and animal phenotypes have increased. Hernandez-Sanabria et al. (12) and Carberry et al. (11) mentioned that specific bacterial clusters are linked with feed efficiency traits and residual feed intake (RFI) in beef cattle. To further understand this relationship, Li et al. (13) delved into the metatranscriptome of the rumen of beef steers differing in RFI. They found that the relative abundance of *Lachnospiraceae*, *Veillonellaceae*, and the uncultured group p-2534-18B5 differed between low RFI (efficient) and high RFI (inefficient) steers. Moreover, the microbiome of efficient steers was metabolically more active and had higher cell adaptability to dietary changes and low-quality diets than inefficient steers. In sheep, Ellison et al. (14) investigated a model for predicting the feed efficiency status of 20 ewes using their rumen bacterial profiles, which included nine species of *Prevotella*, *Pseudobutyrivibrio*, *Schwartzia*, and *Treponema*. The ewes’ feed efficiency status was successfully predicted when all nine bacterial species were included in the model. In dairy cattle, Danielsson et al. (15) found that the relative abundance of Proteobacteria was higher in low CH_4_-emitting cows than in high CH_4_-emitting cows. Conversely, the relative abundance of Actinobacteria was higher in high CH_4_-emitting cows than in low CH_4_-emitting cows. Moreover, the operational taxonomic units (OTU) of *Succinivibrionaceae* and *Ruminococcus* were more abundant in low CH_4_-emitting cows, whereas OTUs of *Fibrobacter*, *Bacteroidales*, and *Bifidobacteriaceae* were more abundant in high CH_4_-emitting cows (15). More recently, Xie et al. (16) and Stepanchenko et al. (17) revealed that the bacterial profiles in the rumen of dairy cows differing in CH_4_-emitting potential were different. Thus, the bacterial profiles of animals differing in feed efficiency status, RFI, and CH_4_-emitting potential differ and cluster differently based on a specific phenotype. Although studies linking ruminal microbial signatures to phenotype have been reported, collecting and analyzing rumen samples in commercial herds is costly and logistically challenging. Identifying alternative phenotypic markers that are more affordable and practical to implement and can serve as proxies for selecting animals with a low CH_4_-yield phenotype is essential.

Given the association between ruminal bacteria and animal phenotypes and the mounting interest in finding suitable phenotypes for identifying animals with low CH_4_-emitting potential, rumination time (RT) has emerged as a potential behavioral trait for identifying phenotypes. RT can be continuously monitored using wearable precision livestock technologies, such as ear tags or neck collars equipped with accelerometers (18, 19). These devices allow for large-scale, on-farm data collection without interfering with normal animal behavior, making them a feasible alternative for identifying phenotypes in commercial herds (20). The RT trait has a heritability ranging between 0.17 and 0.34 (21), indicating that RT is a moderately heritable trait that can serve as a proxy for identifying animals with low CH_4_-emitting potential. Indeed, Mikuła et al. (22), López-Paredes et al. (21) and Castaneda et al. (23) found that cows with a long RT had low EME, which can be selected for permanent and cumulative CH_4_ emission reductions over generations (24). Given its ease of data collection, integration into herd management systems, and correlation with CH₄ emissions, RT measured by wearable sensors may be a scalable and cost-effective tool for identifying low-CH₄ emitting animals in commercial settings.

The rumen microbiome is widely recognized as being linked to the CH_4_-yield phenotype, as demonstrated in dairy cattle (17), beef cattle (25), and sheep (26). Notably, we also established that RT behavior is associated with CH_4_ yield in dairy cattle (23). This raises the question: Is the RT-CH_4_-yield relationship mediated by the rumen microbiome? If so, can bolus and fecal samples also serve as proxies for this phenotype? Rumen samples, typically obtained via stomach tubing (27), require specialized equipment, trained personnel, and animal restraint, limiting their practicality on commercial farms. In contrast, bolus and fecal samples offer viable, less invasive alternatives. Bolus samples can be collected from regurgitated material during rumination, while fecal samples are readily obtained from the rectum, requiring minimal training and no restraint. More invasive methods, such as rumenocentesis and cannulation, pose risks and are impractical for large-scale implementation. Despite this progress, key knowledge gaps remain. First, the link between bacterial and archaeal profiles in cows with differing RT and their connection to CH_4_ yield via the rumen microbiome has not been investigated. Second, it is unknown whether microbial profiles from rumen, bolus, and fecal samples can complement phenotypic data to identify low-CH_4_-emitting animals. We hypothesize that cows with distinct RT phenotypes harbor unique bacteria-archaea cohorts, with significant differences in the microbiomes of rumen contents, bolus, and fecal samples. Furthermore, we propose that these microbial variations, in combination with RT behavior, provide a complementary approach for identifying low-CH_4_-emitting animals.

This study aims to (i) characterize the 16S rDNA-based bacterial profiles and the rumen microbiomes of “low rumination” (LR) and “high rumination” (HR) dairy cows; (ii) investigate whether the bolus or fecal microbiome can serve as a reliable proxy for distinguishing LR and HR phenotypes, and (iii) assess the presence of diurnal rhythmic patterns in the microbiota of rumen, bolus, and fecal samples from LR and HR cows.

## MATERIALS AND METHODS

The protocols for animal handling and care were reviewed, approved, and conducted according to the guidelines of The University of Pennsylvania Institutional Animal Care and Use Committee under application number 80614. The committee also reviewed and approved the experiment and all the procedures involving animals. This study officially started on 8 August 2022 and ended on 24 September 2022.

### Sample collection

In our study of Castaneda et al. (23), we investigated whether the RT and eating time (ET) phenotypes are associated with the CH_4_ yield phenotype, aiming to identify lactating dairy cows with low CH_4_-emitting potential. Briefly, in our study, we selected a group of 49 cows in early to mid-lactation within 150 days in milk. From this group, we selected 20 cows, which, based on their RT, were allocated into the clusters “LR” and “HR”, containing 10 cows each. Thus, this is a follow-up study to reveal the microbiomes of rumen, bolus, and fecal samples of LR and HR cows.

All Holstein lactating cows in our university herd were equipped with AfiCollars (Afimilk, Kibbutz Afikim, Israel), which use accelerometers to continuously record rumination activity and eating behavior by detecting jaw movements associated with cud chewing. The data were automatically transmitted to a central database, processed, and analyzed to quantify individual cow rumination patterns. Rumination data were retrieved at hourly intervals for further analysis, as described in Castaneda et al. (23). Before starting the experiment, the cows were assigned and trained to eat in individual tubs (American Calan Inc., Northwood, NH) for 2 weeks. Subsequently, the cows were trained and acclimated to the GreenFeed System (C-Lock Inc., Rapid City, SD) for another 2 weeks. After this training and acclimation period, the cows were sampled at (d1) 0 h, (d2) 2 h, (d3) 8 h, and (d4) 14 h post-feeding in week 5 of the experimental period. This sampling schedule was designed to have a reasonable foundation of a 24 h CH_4_ emission cycle to couple the bacterial-archaeal profiles of LR and HR cows. The cows’ enteric gas emissions (CO_2_, CH_4_, and H_2_) were measured from all cows individually. During sampling, each cow was allowed to access the GreenFeed System for 5 minutes, followed by a 2-minute waiting gap to avoid mixing the current breath sample with the next. When not sampled, the cows had free access to the GreenFeed System at all times (23). Subsequently, rumen, bolus, and fecal samples were collected. Bolus samples were obtained directly from the cow’s mouth when ruminating, whereas fecal samples were obtained by inserting the hand into the cow’s rectum.

The rumen samples were collected using a stomach tube method as described in our previous paper (28). To collect the sample, the head of the animal was restrained, and ruminal fluid was collected by passing the tubing using an oral speculum down the esophagus into the rumen. The tubing was gently pushed through the rumen mat to collect ruminal contents. Approximately 200 cm of the stomach tube was inserted into the rumen to extract a representative ruminal fluid sample. Whole digesta contents were collected from the rumen and separated into liquid and solid fractions using four layers of cheesecloth (27). We have previously analyzed both solid (29) and liquid (28) samples for bacterial diversity and identified that the liquid fraction showed more intra-day variations concerning water intake and the liquid content in the planktonic phase, whereas the solid fraction was more resilient to intra-day perturbations and showed consistency throughout the day, suggesting the solid fraction is reflective of microbial activity than the liquid fraction (27–29). The liquid fraction underwent volatile fatty acids (VFAs) analysis through high-performance liquid chromatography (HPLC), whereas the solid fraction underwent DNA extraction, PCR amplification, sequencing, and metagenomics analysis.

### DNA extraction, PCR amplification, sequencing, library preparation, and metagenomics

The genomic DNA was extracted from the rumen, bolus, and fecal samples and purified following the “repeated bead beating plus column” (RBB + C) method (30) and using the QIAmp Fast DNA Stool Mini Kit (Qiagen Sciences; Germantown, MD, USA). The V1–V2 regions of the bacterial 16S rRNA gene of each sample were then PCR-amplified in triplicate using the Accuprime Taq DNA Polymerase System (Invitrogen, Carlsbad, CA, United States) (31). The bacterial-specific primers used were F27 (5′-AGAGTTTGATCCT GGCTCAG-3′) and R338 (5′-TGCTGCCTCCCGTAGGAGT-3′). This primer pair was barcoded with a unique 12-base error-correcting Golay code for multiplexing (32). The thermal cycling conditions through which the samples were amplified were followed based on the methodology described by Pitta et al. (31). The triplicate amplicon products of each sample were pooled and quantified using the Spectramax M2e microplate reader (Molecular Devices, San Jose, CA, USA). Finally, the generated amplicons from each sample were pooled in an equimolar concentration and purified using Agencourt AMPure XP Beads (Beckman-Coulter; Indianapolis, IN, USA). The sequencing of rumen, bolus, and fecal samples collected at 0, 2, 8, and 14 h post-feeding was conducted at the PennCHOP Microbiome Core, University of Pennsylvania, using the MiSeq Illumina Platform (Illumina Inc., San Diego, CA, USA). Last, for metagenomics analysis, the DNA of the rumen, bolus, and fecal samples collected at 2 h and 8 h post-feeding was prepared for whole-genome shotgun sequencing using the Nextera DNA Library Prep kit (Illumina, San Diego, CA, USA). The library (tight insert size of 250 bp for high-throughput sequencing from both ends by 2 × 150 bp) was sequenced on an Illumina HiSeq 2500 at the Center for Host-Microbe Interactions and the PennCHOP Microbiome Core of the University of Pennsylvania School of Veterinary Medicine.

### Bioinformatics analysis

We processed bacterial 16S rRNA sequencing data using the QIIME2 version 2020.6 (33) pipeline. The steps included sorting reads by sample (demultiplexing), trimming both forward and reverse reads to 230 nucleotides, and merging paired reads. Unique DNA sequences, called amplicon sequence variants (ASVs), were identified using the DADA2 plugin (33). Sequences were aligned with MAFFT (34), and a phylogenetic tree was built using FastTree 2 (35). Taxonomic assignments were made by matching ASVs to the Greengenes reference database (v37) (36) with a naive Bayes classifier (37). Alpha diversity (observed ASV and Shannon diversity) and beta diversity (weighted and unweighted UniFrac) metrics were calculated using the “qiime diversity” after normalizing the data to a sample depth of 5,847 reads per sample. For shotgun metagenomics, reads were filtered and trimmed with Trimmomatic (v0.36) (38). Host genome sequences (cow genome ARS-UCD1.2/bosTau9) were removed using Bowtie2 (39). Taxonomic profiles were generated using Kraken2 (40) with all the complete bacterial, archaeal, and viral genomes in the National Center for Biotechnology Information’s RefSeq, and Bracken (v2.0) (41) was used to estimate the species abundances. Functional gene and pathway analysis was done by aligning quality-filtered reads to the KEGG (42) database using DIAMOND (v0.9.24) (43), followed by quantifying KEGG Orthologous groups with HUMAnN2 (44). Hydrogenases were classified by aligning reads to a hydrogenase database (45) using DIAMOND (43). All bioinformatics methods were run with default settings unless otherwise specified.

### Statistical analysis

Animal response variables were analyzed using the PROC MIXED procedure of the SAS statistical software Version 9.4 TS1M8 (9.4 M8; SAS Institute Inc.). The response variables were enteric gas emissions (CO_2_, CH_4_, and H_2_), production parameters (CH_4_ efficiency, CH_4_ intensity, dry matter intake [DMI], dry matter [DM] of refusals, milk yield [MY], and feed efficiency), phenotypic responses (RT and ET), and fermentation parameters (total and individual VFA). The lsmeans procedure was used to estimate the means and SEs of the mentioned variables. A repeated measures arrangement was performed to account for the effect of sampling hour. Cow group (LR and HR) and CowID were deemed fixed and random effects, respectively. The microbiome data were analyzed in R (version 4.2.1). A mixed linear model (lme4 package in R) was used to assess alpha diversity metrics (observed ASV and Shannon diversity), taxonomy data (16S rDNA and metagenomics), and functional genes or enzymes. Fixed effects included group, sampling hour, and their interaction, with CowID as a random effect. Beta diversity was analyzed using permutational multivariate analysis of variance (PERMANOVA) (vegan package in R) with weighted and unweighted UniFrac matrices. The same model was applied, with 999 permutations and CowID as a blocking factor. The data were log transformed for the mixed linear models to meet model assumptions. *P*-values for multiple comparisons were adjusted using the Tukey method with the emmeans package in R. We used Spearman correlation to explore relationships between individual VFAs and bacterial populations identified by 16S rRNA sequencing. Differences in rumination and eating times between LR and HR groups were analyzed using generalized additive models.

## RESULTS

### Enteric gas emissions, feeding behavior, production efficiency, and phenotypic traits

Based on our previous report (23), we established a method to identify cows with a distinct RT phenotype that showed differences in EME. Differences in EME, feeding behavior, production efficiency, and VFA between LR and HR are summarized in Table 1. The daily average EME (mean ± SE) of HR cows (404 ± 6.04 g/day) was significantly lower (*P* = 0.003) than that of LR cows (430 ± 6.27 g/day). CO_2_ emissions were significantly lower (*P* < 0.001) in LR cows (14,252 ± 202 g/day) compared to HR cows (15,498 ± 194 g/day). H_2_ emissions were slightly lower in HR cows (3.54 ± 0.13 g/day) than in LR cows (3.84 ± 0.13 g/day). To estimate the production efficiency of LR and HR cows, EME was compared against DMI and MY for CH_4_ efficiency (g CH_4_/kg DMI) and intensity (g CH_4_/kg milk), respectively. The CH_4_ efficiency and intensity of LR cows (23.6 ± 0.64 and 13.9 ± 0.30) differed significantly (*P* < 0.001) from HR cows (17.1 ± 0.64 and 10.3 ± 0.30). The DMI and MY of HR cows were 24.6 ± 0.28 and 40.6 ± 0.47, whereas for LR cows, they were 20.9 ± 0.28 and 35.1 ± 0.47. RT and ET showed significant differences (*P* < 0.001) between LR and HR cows throughout the day. HR cows ruminated for 494 ± 31.6 min/day and ate for 261 ± 15.9 min/day, while LR cows ruminated for 400 ± 24.4 min/day and ate for 175 ± 10.7 min/day.

**TABLE 1:** Enteric gas emissions, production efficiency, feeding behavior, and rumen fermentation variables phenotypic group of LR and HR Holstein cows

Variable	Phenotype group^*a*^		*P*-value,^*b*^ LR vs HR
	LR	HR	
Enteric gas emissions			
CH_4_ (g/day)	430 ± 6.27	404 ± 6.04	0.003
CH_4_ per DMI (g/kg)	23.6 ± 0.64	17.1 ± 064	<0.001
CH_4_ per MY (g/kg)	13.9 ± 0.30	10.3 ± 0.30	<0.001
CO_2_ (g/day)	14,252 ± 202	15,498 ± 194	<0.001
CO_2_ per DMI (g/kg)	798 ± 22.45	630 ± 22.45	<0.001
CO_2_ per MY (g/kg)	479 ± 10.2	400 ± 10.2	<0.001
H_2_ (g/day)	3.84 ± 0.13	3.54 ± 0.13	0.095
Production parameters			
DMI^*c*^ (kg/day)	20.9 ± 0.28	24.6 ± 0.28	<0.001
DM of refusals (%)	52.5 ± 0.67	49.0 ± 0.67	<0.001
MY^*c*^ (kg/day)	35.1 ± 0.47	40.6 ± 0.47	<0.001
Feed efficiency^*d*^ (kg/kg)	1.73 ± 0.04	1.72 ± 0.04	0.868
Phenotypic responses			
RT (min/day)	400 ± 24.4	494 ± 31.6	<0.001
ET (min/day)	175 ± 10.7	261 ± 15.9	<0.001
Fermentation parameters			
Total VFA (mol %)	88.02 ± 2.095	86.70 ± 2.108	0.658
Acetic (mol %)	59.59 ± 0.3038	56.51 ± 0.3056	<0.001
Propionic (mol %)	22.86 ± 0.2727	25.82 ± 0.2744	<0.001
Isobutyric (mol %)	1.59 ± 0.0305	1.55 ± 0.0307	0.371
Butyric (mol %)	13.09 ± 0.1465	13.00 ± 0.1474	0.665
Isovaleric (mol %)	1.38 ± 0.0599	1.43 ± 0.0603	0.031
Valeric acid (mol %)	1.49 ± 0.0427	1.70 ± 0.0429	0.001
Acetic (mM)	52.52 ± 1.2884	49.15 ± 1.2963	0.067
Propionic (mM)	20.15 ± 0.5638	22.31 ± 0.5672	0.007
Isobutyric (mM)	1.37 ± 0.0261	1.29 ± 0.0262	0.048
Butyric (mM)	11.46 ± 0.2857	11.28 ± 0.2874	0.652
Isovaleric (mM)	1.19 ± 0.0496	1.23 ± 0.0499	0.586
Valeric (mM)	1.32 ± 0.0453	1.43 ± 0.0456	0.104

^*a*^ Phenotypic groups were LR and HR. Values are expressed 6 as mean ± SE.

^*b*^ The *P*-values for all variables were derived using the Proc Mixed fixed-effect model in SAS statistical software, with the exception of phenotypic responses (RT and ET), for which a generalized linear mixed-effects model was employed.

^*c*^ DMI and MY data were obtained from the last 3 weeks before sampling inclusive.

^*d*^ Feed efficiency = MY ÷ DMI.

### Sequencing information

A total of 13,375,332 raw paired reads were generated from a total of 229 samples (rumen: 75; bolus: 76; fecal: 78), with an average of (mean ± SD) 58,408 ± 19,948 reads per sample (,Table S1). This produced a total of 7,321,872 ASV. Representative sequences from the ASV were assigned to 24 distinct phyla, 2 of which could not be classified. A total of 369 genera categories were observed in this study, of which 216 (59%) were classified to genus level, 151 (41%) were unclassified to either phylum, class, order, or family, and 2 genera were unclassified to bacterial genus. For metagenomics, the Illumina HiSeq generated a total of 1,992,813,754 sequences for 117 samples (rumen: 39; bolus: 39; fecal:39). After quality filtering, approximately 14% of reads were eliminated, resulting in a total of 1,708,446,278 quality filtered reads with an average of 14,602,105 ± 3,673,033 paired reads per sample (Table S2). A taxonomy assignment with Kraken2 revealed that approximately 95% and 5% of sequences were assigned to bacteria and archaea, respectively. Eukaryotes and viruses constituted very small proportions.

### Comparison of the relative abundance, methanogen diversity, and associated methanogenic pathways

**Fig 1:**
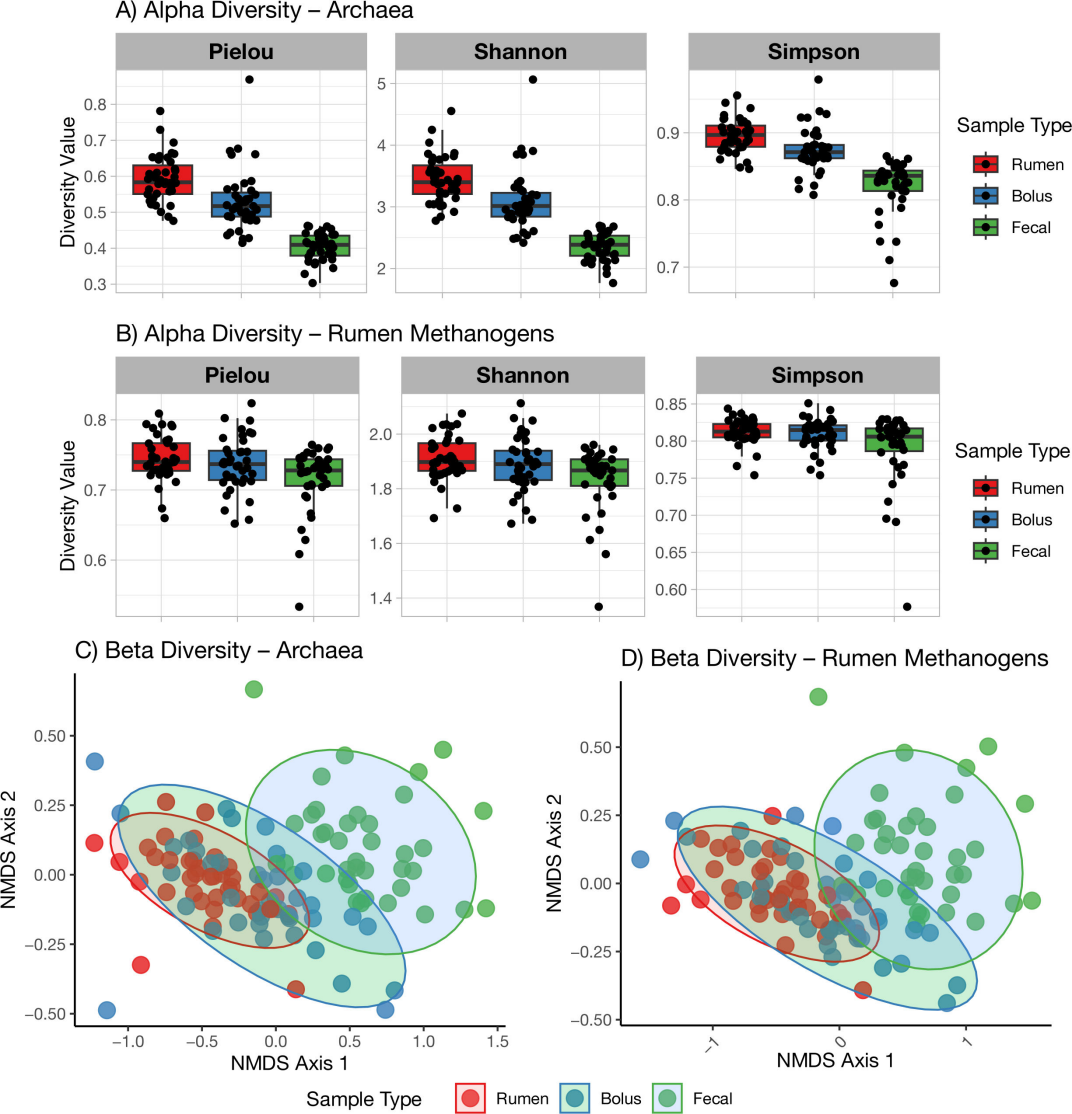
Assessment of rumen archaeal diversity based on metagenomics in ruminating Holstein cows across sample types (bolus, rumen, and feces). (**A and B**) Boxplots showing within-sample diversity (alpha diversity), measured by Pielou’s evenness, Shannon diversity index, and Simpson diversity index, for (**A**) archaea and (**B**) ruminal methanogens. (**C and D**) NMDS plots based on Bray-Curtis distances illustrating archaeal community composition, stratified by sample type, for (**C**) archaea and (**D**) ruminal methanogens.

For alpha diversity, across bolus, rumen, and fecal microbiomes, archaea and methanogenic species were identified using the Kraken pipeline on metagenomic data (Fig. 1; Table 2; Data set S2A through C). The rumen samples revealed the most diverse archaeal species compared to bolus and fecal samples (Fig. 1; *P* < 0.05; Shannon; Simpson; and Pielou’s evenness). As expected, bolus samples were similar to rumen samples for methanogen diversity (Fig. 1B; *P* > 0.5; Shannon; Simpson; and Pielou’s evenness). For beta diversity, it was clear that rumen samples were similar to bolus samples based on the overlap (non-metric multidimensional scaling [NMDS]) between the rumen and bolus clusters (Fig. 1C; *R*^2^ = 0.010; *P* = 0.68; Fig. 1D; PERMANOVA *R*^2^ = 0.009; *P* = 0.73). There was limited overlap (NMDS) between fecal and the other two sample types, indicating that fecal methanogen community composition may be different from rumen and bolus for the type of archaea populations distributed across the samples (Fig. 1C and D). To differentiate methanogens from total archaea, we selected previously reported methanogens and compared them between the three sample types (Table 2). Although the dominant methanogens were identified across the three sample types, there are differences in the relative abundance of individual methanogens. The CO_2_-reducing methanogens were dominant, which include species of *Methanobrevibacter* such as *M.* YE315, *Methanobrevibacter ruminantium*, *Methanobrevibacter olleyae*, *Methanobrevibacter millerae*, *Methanobrevibacter smithii*, *M.* TLL-48-HuF1, and *M.* AbM4. Following the CO_2_-utilizing methanogens were methanol-utilizing methanogens, such as *Methanosphaera* ISO3-F5, *Methanosphaera* BMS, and *Methanosphaera stadtmanae*. In rumen samples, among the most abundant methanogens, *M.* YE315 was higher, whereas *Methanosphaera* ISO3-F5 and *Methanosphaera stadtmanae* were lower in LR compared to HR cows. There were not many significant differences in sampling hours for the rumen samples. In fecal samples, *M.* YE315 was higher in LR compared to HR cows, whereas in bolus samples, *Methanosphaera* ISO3-F5 and *Methanosphaera stadtmanae* were lower in LR compared to HR cows.

**TABLE 2:** Mean relative abundance (%) of methanogenic populations in bolus, rumen, and fecal samples from LR and HR Holstein cows at 2 h and 8 h sampling intervals^*a*^

Sample	Methanogen	LR			HR			*P*-value		
		2 h	8 h	SEM	2 h	8 h	SEM	Grp	Hour	Grp:h
Bolus	*Methanobrevibacter ruminantium*	18.99	17.42	0.86	20.94	17.86	0.70	0.172	0.464	0.429
	*Methanobrevibacter olleyae*	19.85	18.75	1.38	19.06	16.80	1.25	0.894	0.691	0.623
	*Methanobrevibacter* sp. YE315	16.99	18.48	1.00	14.03	14.48	1.21	0.189	0.139	0.977
	*Methanobrevibacter millerae*	10.79	12.31	0.67	11.62	16.51	1.42	0.517	0.092	0.445
	*Methanobrevibacter smithii*	3.63	4.18	0.20	3.90	4.19	0.13	0.281	0.014	0.333
	*Methanobrevibacter* sp. TLL-48-HuF1	3.11	3.60	0.20	3.54	3.60	0.13	0.109	0.015	0.141
	*Methanosphaera* sp. ISO3-F5	1.24	2.09	0.13	1.77	2.69	0.17	0.006	0.000	0.513
	*Methanobrevibacter* sp. AbM4	1.38	1.31	0.14	1.71	1.47	0.10	0.092	0.899	0.115
	*Methanosphaera* sp. BMS	0.66	1.17	0.09	0.77	1.08	0.06	0.297	0.000	0.105
	*Methanobrevibacter arboriphilus*	0.69	0.70	0.06	0.89	0.82	0.05	0.046	0.517	0.210
	*Methanosphaera stadtmanae*	0.41	0.69	0.04	0.53	0.88	0.06	0.051	0.000	0.550
	*Methanomethylophilus alvi*	0.45	0.33	0.09	0.35	0.31	0.04	0.623	0.558	0.803
	Uncultured *Methanoregula* sp*.*	0.25	0.26	0.04	0.20	0.26	0.02	0.555	0.620	0.702
Rumen	*Methanobrevibacter ruminantium*	16.49	17.20	0.56	18.74	18.15	0.70	0.102	0.330	0.277
	*Methanobrevibacter olleyae*	16.26	17.39	1.15	16.38	15.27	1.00	0.987	0.299	0.223
	*Methanobrevibacter* sp. YE315	17.01	18.78	0.94	12.87	14.47	1.12	0.054	0.164	0.950
	*Methanobrevibacter millerae*	9.77	11.07	0.51	10.09	12.08	0.83	0.751	0.135	0.856
	*Methanobrevibacter smithii*	3.63	4.07	0.16	3.64	3.92	0.09	0.766	0.033	0.517
	*Methanobrevibacter* sp. TLL-48-HuF1	3.21	3.45	0.15	3.22	3.44	0.09	0.783	0.117	0.857
	*Methanosphaera* sp. ISO3-F5	1.34	1.76	0.10	1.98	2.11	0.16	0.009	0.058	0.256
	*Methanobrevibacter* sp. AbM4	1.20	1.26	0.10	1.50	1.50	0.09	0.095	0.151	0.300
	*Methanosphaera* sp. BMS	0.82	1.05	0.07	0.83	0.94	0.05	0.974	0.069	0.534
	*Methanobrevibacter arboriphilus*	0.62	0.71	0.05	0.76	0.90	0.05	0.114	0.010	0.708
	*Methanosphaera stadtmanae*	0.46	0.59	0.03	0.65	0.72	0.06	0.039	0.091	0.494
	Uncultured *Methanoregula* sp.	0.40	0.32	0.03	0.40	0.36	0.03	0.991	0.137	0.534
Feces	*Methanobrevibacter millerae*	27.25	22.86	1.58	25.37	25.01	2.82	0.515	0.145	0.283
	*Methanobrevibacter* sp. YE315	26.83	24.54	0.87	21.40	19.44	1.81	0.036	0.072	0.631
	*Methanobrevibacter ruminantium*	12.71	13.36	0.77	16.87	15.72	1.15	0.106	0.467	0.477
	*Methanobrevibacter olleyae*	10.98	13.61	0.98	13.78	14.10	1.37	0.496	0.139	0.452
	*Methanobrevibacter smithii*	5.79	5.91	0.10	5.16	5.32	0.24	0.051	0.679	0.523
	*Methanobrevibacter* sp. TLL-48-HuF1	4.88	4.83	0.12	4.44	4.38	0.23	0.149	0.801	0.768
	*Methanosphaera* sp. ISO3-F5	2.12	2.18	0.08	1.97	2.40	0.14	0.399	0.687	0.139
	*Methanobrevibacter* sp. AbM4	1.59	1.50	0.13	1.71	1.54	0.10	0.508	0.744	0.593
	*Methanosphaera* sp. BMS	0.99	1.34	0.08	0.86	1.08	0.04	0.206	0.000	0.376
	*Methanobrevibacter arboriphilus*	0.78	0.82	0.05	0.87	0.86	0.04	0.327	0.491	0.622
	*Methanosphaera stadtmanae*	0.60	0.76	0.03	0.58	0.84	0.05	0.688	0.005	0.207

^*a*^ Grp: Group.

In the rumen samples, we quantified gene copies of enzymes associated with H_2_-utilizing pathways using metagenomic data (Table S3A through C). The CO_2_-reducing pathway was identified as the predominant methanogenic pathway, while the methanol- and methylamine-utilizing pathways were less prominent. Although the sample size was limited, numerical differences in gene copy abundances were observed between the LR and HR groups and between the 2 h and 8 h post-feeding (Table S3A). Gene copies associated with enzymes involved in the methanol-utilizing pathway were notably higher in HR cows compared to LR cows. Moreover, *Methanosphaera* species and their corresponding gene copies were consistently more abundant in HR cows than in LR cows.

Gene copies of enzymes involved in the various methanogenesis pathways of bolus samples (Table S3) were found to closely resemble those in rumen samples, indicating that bolus samples can serve as a reliable proxy for rumen samples when assessing methanogen diversity and methanogenesis pathways. In fecal samples (Table S3C), gene copies of enzymes associated with these pathways exhibited similar patterns to those observed in rumen and bolus samples. However, the copy numbers were significantly higher in fecal samples compared to the other two sample types.

### Changes in methyl coenzyme M reductase and tetrahydromethanopterin S-methyltransferase enzymes

The enzyme methyl coenzyme M reductase (MCR; EC: 2.8.4.1) catalyzes CH_4_ formation in all methanogens. In the current study (Table S3A, C, and E), the copy number of the genes encoding for EC: 2.8.4.1 was among the most abundant genes but did not show a significant difference in the rumen (LR 311.1 vs HR 312.2), bolus (LR 391.2 vs HR 386.2), and fecal samples (LR 988.6 vs HR 932.8) at 2 h post-feeding between LR and HR cows. However, the genes responsible for tetrahydromethanopterin S-methyltransferase (MTR; EC: 7.2.1.4) that enable the transfer of the methyl group to MCR showed a numerical difference of approximately 10% between LR and HR cows in both rumen (LR 757.9 vs HR 691.1) and bolus samples (LR 822.7 vs HR 731.9) at 2 h post-feeding. Differences in EME observed in this study by about 6% are accompanied by differences in the MCR and MTR enzymes. However, the high efficiency and lower CH_4_ intensity observed in HR cows compared to LR cows were based on the high DMI and high MY, respectively.

### Comparison of alternative H_2_-utilizing sinks

**Fig 2:**
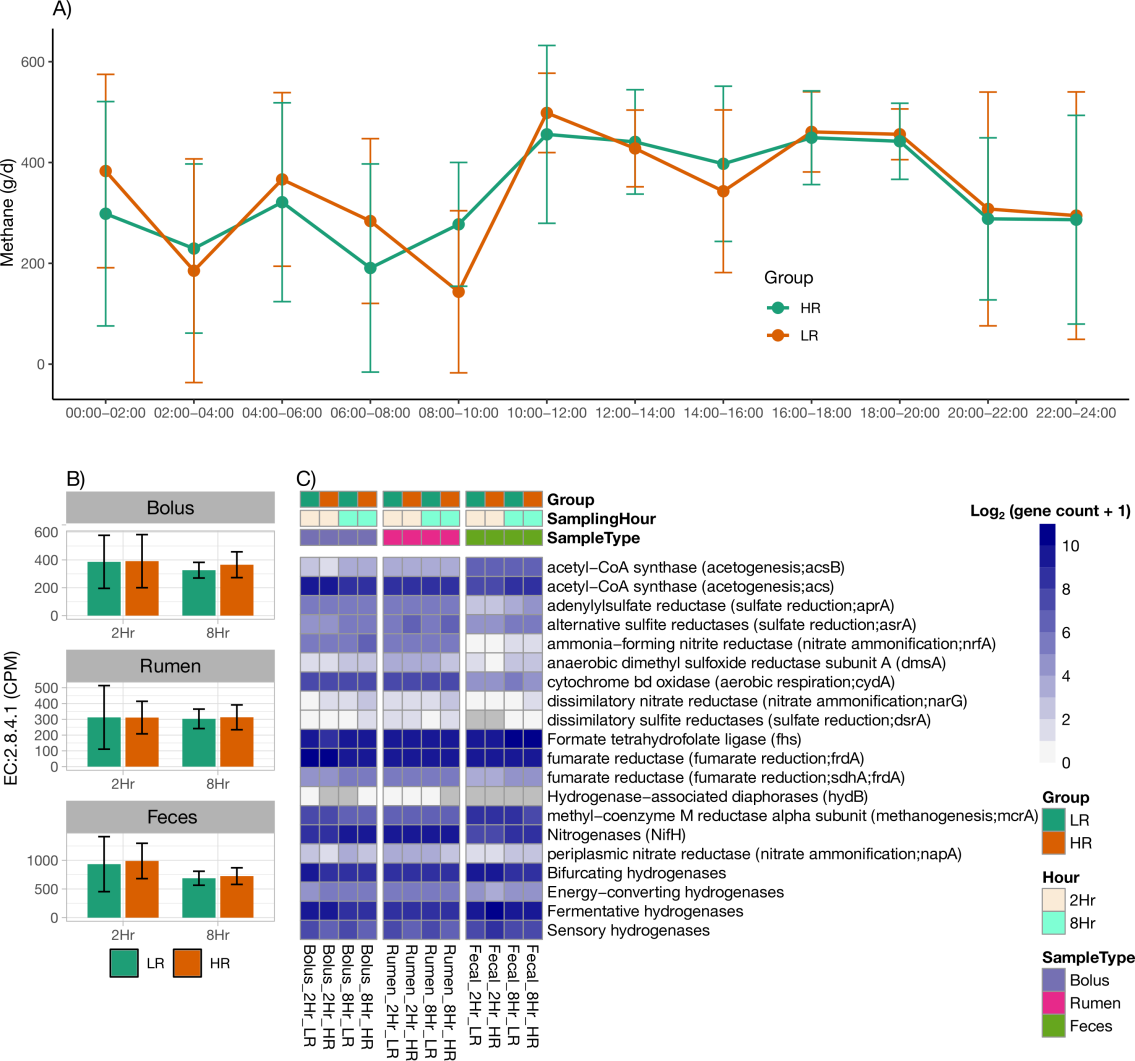
EMEs, methanogenic gene abundance, and hydrogen (H_2_)-utilizing pathways in LR and HR Holstein cows across sample types (bolus, rumen, and feces). (**A**) Circadian patterns of mean enteric CH_4_ emissions in LR and HR cows, measured at 2 h intervals over a 24 h cycle. (**B**) Mean abundance of gene counts (copies per million [CPM]) for the enzyme EC:2.8.4.1 (MCR), a key enzyme in methanogenesis, measured at 2 h and 8 h sampling intervals across the three sample types. (**C**) Heatmap of log-transformed gene counts (CPM) for enzymes involved in alternative H_2_-utilizing pathways, measured at 2 h and 8 h sampling intervals across the three sample types. Error bars represent the SD.

We identified potential H_2_-utilizing pathways in the rumen and compared the gene copies of the most important enzymes with each of the H_2_-utilizing pathways (Table S3D through F; Fig. 2C). The predominant H_2_ sink was methanogenesis, in which methyl coenzyme reductase *mcrA* was marginally lower in HR cows compared to LR cows (Fig. 2B). Although the *mcrA* gene copies were only slightly lower, alternative H_2_-utilizing pathways, such as sulfate reductase (*dsrAB*), fumarate reductase (*frdABCD*), nitrate reductase (*napA*), and ammonia-forming nitrite reductase (*nirS*), were numerically higher in HR cows compared to LR cows. The bolus samples showed similar patterns with increases in gene copies of enzymes such as sulfate reductase, fumarate reductase, nitrate reductase, and ammonia-forming nitrite reductase in HR cows, which showed a trend (*P* < 0.09) compared to LR cows. In fecal samples, gene copies of acetyl-CoA synthetase were much higher compared to bolus or rumen samples, whereas all other alternative pathways except *mcrA* and fumarate reductase were lower, suggesting that acetogenesis is a predominant sink in the lower gut microbiome. Overall, it can be inferred that gene copies for alternate pathways for utilizing H_2_ may be upregulated in HR cows compared to LR cows when the latter are dominated by methanogenesis pathways as the predominant H_2_ sink.

### Comparison of the 16S rDNA-based bacterial community

**Fig 3:**
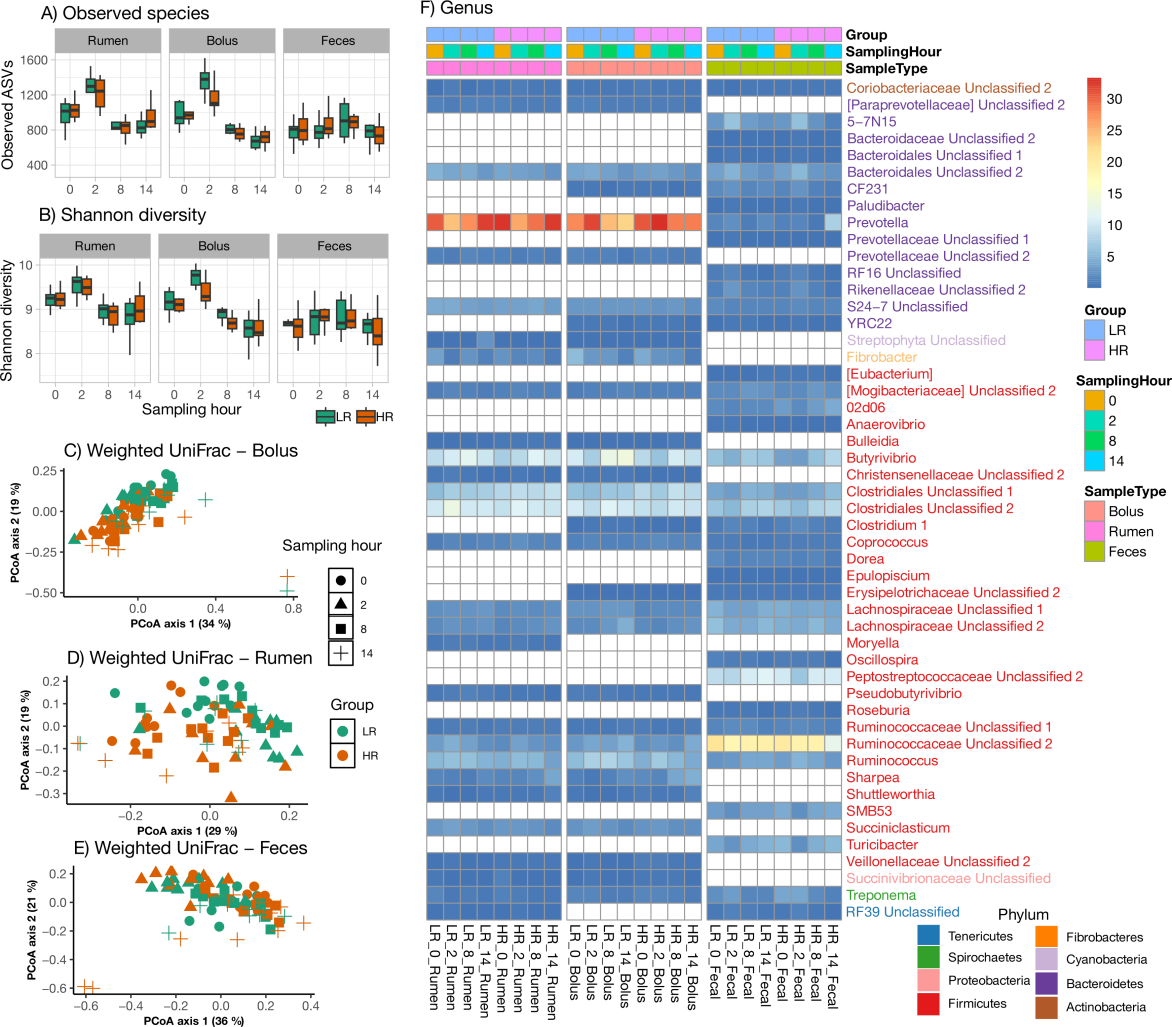
Assessment of rumen 16S rDNA bacterial diversity and composition in LR and HR Holstein cows across sample types (bolus, rumen, and feces) at 0, 2, 8, and 14 h sampling intervals. (**A and B**) Boxplots illustrating within-sample diversity (alpha diversity) as measured by observed species (**A**) and the Shannon diversity index (**B**), stratified by treatment group (LR vs HR) and sampling hour for each sample type. (**C, D,** and **E**) PCoA plots based on weighted UniFrac distances, depicting bacterial community composition, stratified by treatment group and sampling hour for each sample type. (**F**) Heatmap displaying the relative abundance (%) of the most prevalent bacterial genera, stratified by treatment group and sampling hour for each sample type. Bacterial genera are color coded by their corresponding phylum.

Similar to archaeal alpha and beta diversity, bacterial community composition followed the same pattern (Fig. S1). The ruminal and bolus samples had similar species richness and diversity (Shannon; Faith’s PD; Pielou evenness; *P* > 0.5) and clustered together on principal coordinates analysis (PCoA) plots showing similarity between the two sample types. However, fecal samples were distantly clustered from the bolus and the rumen, indicating the composition of fecal bacteria is different from the bolus and rumen. To determine whether differences exist between LR and HR cows and whether bacterial community composition followed a rhythmic pattern within cows, bolus, rumen, and fecal samples were analyzed for bacterial profiles in samples collected at 0 h, 2 h, 8 h, and 14 h post-feeding on week 5 of the experiment (Fig. 3). For within-sample variation (alpha diversity; Fig. 3A and B), observed species and Shannon diversity indices were used to compare differences between cow groups (LR and HR) and sampling hours (0, 2, 8, and 14 h), along with their interactions. No significant differences were observed between the cow groups across all sample types. However, sampling at 2 h post-feeding showed significantly higher diversity than pre-feeding, 8 h, and 14 h post-feeding in bolus and rumen samples but not in fecal samples (Fig. 3B). This pattern is consistent with the idea that feeding triggers a spike in fermentation due to increased microbial activity at 2 h, which then returns to pre-feeding levels in bolus and rumen samples. In contrast, fecal samples remained consistent in terms of both the number and diversity of bacterial populations across all animals.

Beta diversity analysis using PERMANOVA revealed significant effects of group and sampling hour on bacterial community composition across all sample types, as assessed by both weighted and unweighted UniFrac metrics (Data set 4). For weighted UniFrac, the effect of group was significant for rumen (*R*^2^ = 0.068), bolus (*R*^2^ = 0.064), and fecal (*R*^2^ = 0.023) samples (*P* = 0.001), whereas sampling hour exhibited a stronger influence in all sample types, with *R*^2^ values ranging from 0.164 to 0.216 (*P* = 0.001). The group × sampling hour interaction was not significant (*P* > 0.05), indicating that the effects of group and sampling hour were independent. Similarly, unweighted UniFrac analysis showed significant effects of group (rumen *R*^2^ = 0.057; bolus *R*^2^ = 0.057; fecal *R*^2^ = 0.031; *P* = 0.001) and sampling hour (rumen *R*^2^ = 0.065; bolus *R*^2^ = 0.078; fecal *R*^2^ = 0.071; *P* = 0.001). However, the group × sampling hour interaction remained non-significant across all sample types (*P* > 0.05).

### Changes in the 16S rDNA bacterial populations

The 16S rDNA bacterial communities exhibited variations among cow groups, sample types, and sampling hours (Fig. 3C). In rumen samples, an interaction between cow group and sampling hour was identified for *Bacteroidetes* BF311 (*P* = 0.047), *Bacteroidetes* RF16 Unclassified (*P* = 0.009), Firmicutes *Moryella* (*P* = 0.025), and Tenericutes *Anaeroplasma* (*P* = 0.004). Most of the Bacteroidetes detected, such as Bacteroidetes *Prevotella* (*P* < 0.001) and *Bacteroidetes* YRC22 (*P* = 0.036), and most Firmicutes, such as *Firmicutes* L7A-E11 (*P* = 0.005) and *Firmicutes* p-75-a5 (*P* < 0.001), differed significantly among sampling hours. A fewer number of Bacteroidetes, such as Bacteroidetes *Prevotellaceae* Unclassified 1 (*P* = 0.045) and Bacteroidetes *Bacteroidales* Unclassified 2 (*P* = 0.006), and an even fewer number of Firmicutes, such as Firmicutes *Clostridiales* Unclassified 1 (*P* < 0.001) and Firmicutes *Ruminococcaceae* Unclassified 2 (*P* = 0.007), differed significantly among groups. Firmicutes *Ruminococcaceae* Unclassified 2 (4.48 vs 3.41), Firmicutes *Ruminococcus* (5.90 vs 5.63), and Bacteroidetes RF16 Unclassified (0.062 vs 0.041) were more abundant in LR cows than in HR cows, whereas Firmicutes *Clostridiales* Unclassified 1 (7.04 vs 8.76) were more abundant in HR than in LR cows. Other bacteria, such as Tenericutes *Anaeroplasma* (*P* < 0.001) and Spirochetes *Treponema* (*P* < 0.001), differed significantly between cow groups and among hours, respectively. Bacteroidetes, Firmicutes, and small populations, such as Proteobacteria, Tenericutes, and Spirochetes of bolus and fecal samples, were also affected by cow group and sampling hour similarly to rumen samples.

A spike in the 16S rDNA bacterial communities was observed at 2 h post-feeding in the bolus and rumen samples of both cow groups, although at 8 h and 14 h post-feeding, they returned to pre-feeding levels (Fig. 3C and D). This intraday pattern was more accentuated in bolus samples than in rumen samples but not observed in fecal samples (Fig. 3C), where the 16S rDNA bacterial communities showed minimal fluctuations pre- and post-feeding. Besides this finding, it is also worth noting that changes in the 16S rDNA bacterial communities between LR and HR groups were noted but were more pronounced by sampling hour.

### VFA profiles and their correlation with the 16S rDNA bacterial communities

**Fig 4:**
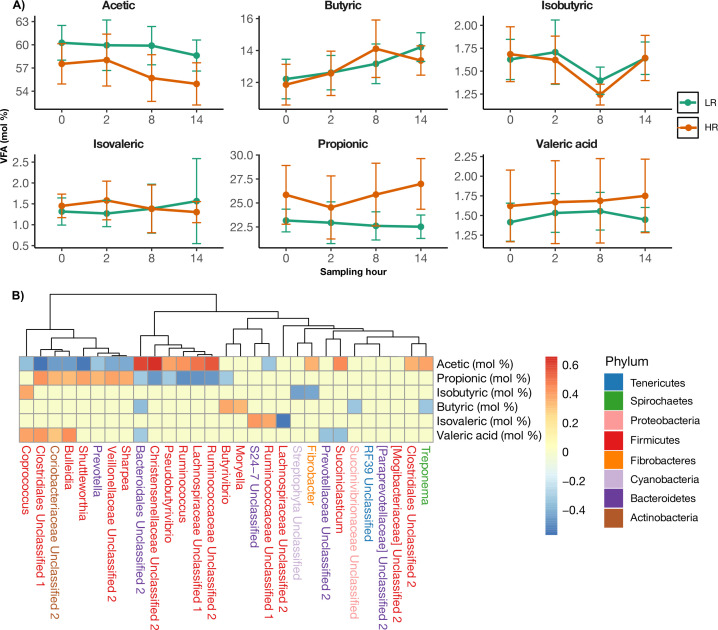
Intraday patterns of rumen fermentation variables (VFA) and their associations with rumen bacterial communities in LR and HR Holstein cows. (**A**) Intraday variations in rumen fermentation variables (mol %) across sampling hours. (**B**) Spearman correlation analysis between rumen bacterial genera (mol %) and rumen fermentation profiles. Bacterial genera are color coded by their corresponding phylum. Abbreviation: mol %, molar proportion. Error bars represent the SD.

The concentrations of total and individual VFAs, expressed in millimoles and molar proportions, are detailed in Table 1. Furthermore, the individual VFAs expressed in molar proportions were calculated and presented in Fig. 4A. The total and individual VFA concentrations exhibited significant variation between cow groups and over the various sampling hours. The molar proportions of acetate were significantly higher (*P* < 0.001) in LR cows compared to HR cows, remaining elevated at all sampling hours and gradually decreasing from 0 h to 14 h post-feeding. A marked difference in the molar proportions of propionate was also noted, with levels consistently higher (*P* < 0.001) in HR cows than in LR cows at all hours. In LR cows, the molar proportions of propionate declined slowly from 0 h to 14 h post-feeding, whereas for HR cows, they decreased from 0 h to 2 h post-feeding, rising significantly from 2 h to 14 h post-feeding. The molar proportions of isovalerate were also significantly higher (*P* < 0.05) in HR cows compared to LR cows, particularly at the 2 h and 14 h post-feeding, albeit similar at 0 h and 8 h post-feeding in both cow groups. Moreover, the molar proportions of valerate were significantly higher (*P* < 0.001) in HR cows than in LR cows, remaining elevated at all hours post-feeding. From 0 h to 14 h, the molar proportions of valerate increased progressively in HR cows, while in LR cows, they rose from 0 h to 2 h, stabilized between 2h and 8 h, and declined from 8 h to 14 h post-feeding. Although no significant differences were observed in the molar proportions of total VFA, isobutyrate, and butyrate between cow groups, these variables were numerically higher in LR cows. These results affirm that the profiles of total and individual VFA concentrations differ between LR and HR cows and among sampling hours, further substantiating the hypothesis that cows with different RT may exhibit variations in fermentation patterns as reflected by the molar proportions of total and individual VFA profiles.

To reinforce the hypothesis that ruminal bacteria and VFA are linked in LR and HR cows, a Spearman rank correlation analysis was conducted between the molar proportions of individual VFA and the predominant bacterial communities, as illustrated in Fig. 4B. In HR cows, a weak inverse relationship (*r* = −0.2) was observed between propionate and species of Firmicutes *Butyrivibrio*, Firmicutes *Pseudobutyrivibrio*, and Bacteroidetes *Bacteroidales* Unclassified 2. Species of *Ruminococcaceae* Unclassified 2, *Lachnospiraceae* Unclassified 1, and *Ruminococcus* of the Firmicutes phyla were also inversely associated (*r* = −0.4) with the molar proportions of propionate in HR cows. It was surprising to note that the opposite effect was observed between these bacteria and the molar proportions of acetate; specifically, as these bacterial species were inversely correlated with the molar proportions of propionate in HR cows, they were positively correlated with the molar proportions of acetate in LR cows. Conversely, Firmicutes *Sharpea*, Firmicutes *Bulleidia*, and Actinobacteria *Coriobacteriaceae* had a moderate positive relationship (*r* = 0.3) with the molar proportions of propionate in HR cows. *Shuttleworthia* and *Clostridiales* Unclassified 1 of the Firmicutes phyla exhibited a moderate positive correlation (*r* = 0.4) with the molar proportions of propionate in HR cows, slightly stronger than the preceding correlation. These findings also contrast with the correlations observed on the molar proportions of acetate in LR cows. Contrarily, these species are positively associated with the molar proportions of propionate in HR cows, where they were inversely correlated with the molar proportions of acetate in LR cows. Thus, bacterial communities correlate with the molar proportions of individual VFA and are concomitantly linked to the RT phenotype, indicating that the bacterial and VFA profiles of LR and HR cows are opposite.

### Genes associated with the propionate and acetate pathways

**Fig 5:**
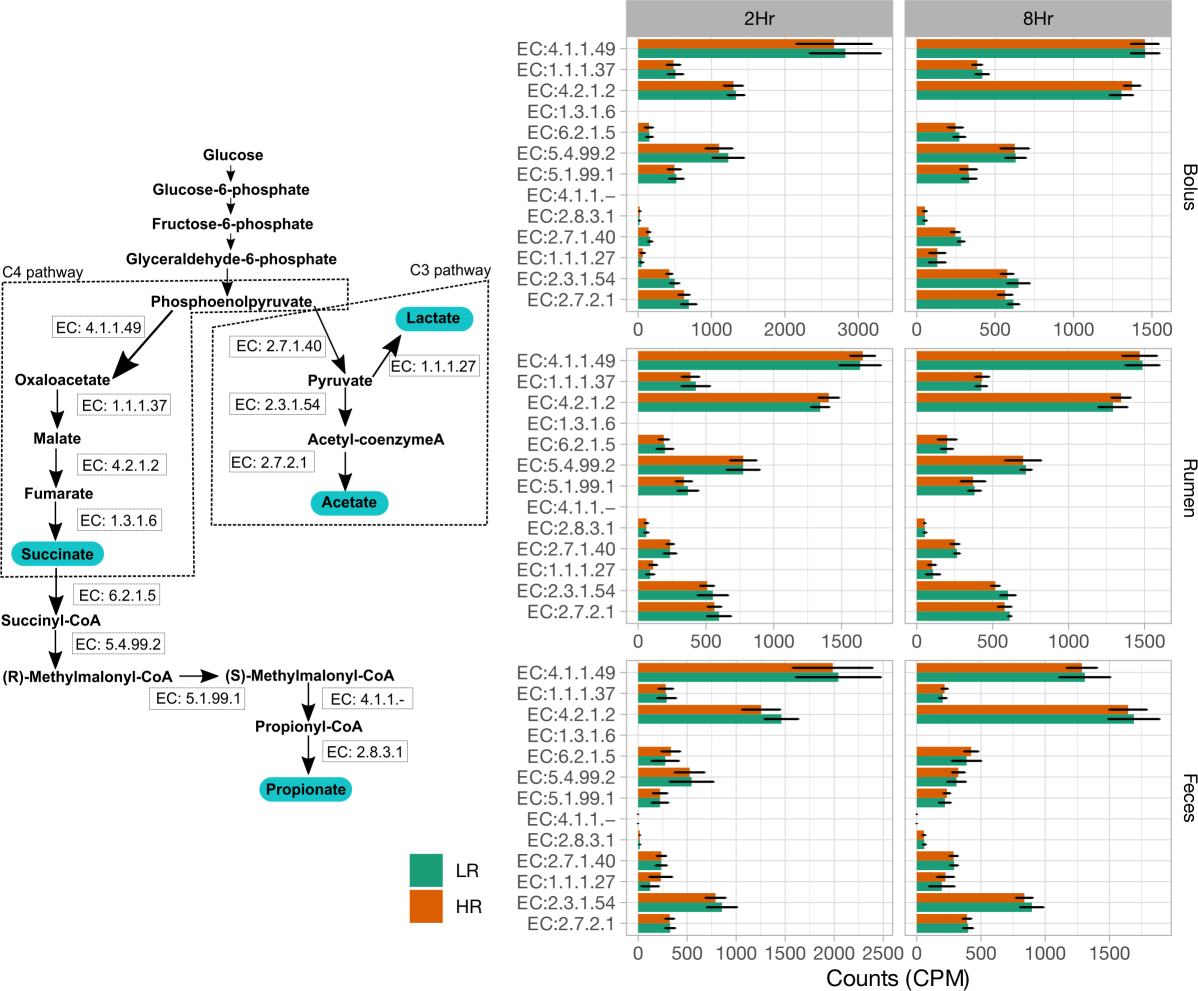
Gene abundance associated with enzymes in the propionate pathway in LR and HR ruminating Holstein cows. Gene abundance, measured in copies per million (CPM), is compared between LR and HR cows across sample types (bolus, rumen, and feces) at 2 h and 8 h sampling intervals. Error bars represent the SD.

Due to the consistently higher propionate levels observed in HR cows compared to LR cows, we performed a metagenomics analysis to examine changes in the propionate pathway within the rumen, bolus, and fecal samples at 2 h and 8 h post-feeding (Fig. 5; Table S3A through C). In rumen samples, there were significantly higher gene copy numbers of the enzyme phosphoenolpyruvate carboxykinase (EC:4.1.1.49) at 2 h for both LR (*P* = 0.001) and HR cows (*P* = 0.005) compared to 8 h post-feeding. The gene copy numbers of the formate C-acetyltransferase enzyme (PFL; EC:2.3.1.54) differed significantly (*P* = 0.01) between LR and HR cows, showing notably higher levels (*P* = 0.014) in HR cows at 8 h post-feeding. In bolus samples, several enzymes displayed significant differences between cow groups and sampling hours. The gene copy numbers of EC:2.7.1.40 and EC:2.3.1.54 were significantly higher at 2 h (*P* = 0.04 and *P* = 0.003) and 8 h post-feeding (*P* = 0.003 and *P* = 0.024) in HR cows compared to LR cows. In fecal samples, significant differences in the gene copy numbers of various enzymes were noted between cow groups and sampling hour. An interaction between the cow group and sampling hour was identified on the gene copy numbers of fumarate hydratase (EC:4.2.1.2; *P* = 0.01) and L-lactate dehydrogenase (EC:1.1.1.27). The gene copy numbers for enzymes EC:4.1.1.49 (*P* < 0.001), EC:1.1.1.37 (*P* = 0.03), and EC:5.4.99.2 (*P* < 0.001) were significantly higher at 2 h compared to 8 h post-feeding in LR cows. Likewise, in HR cows, the gene copy numbers for EC:4.1.1.49 (*P* < 0.001), EC:1.1.1.37 (*P* = 0.004), and EC:5.4.99.2 (*P* < 0.001) were also significantly greater at 2 h compared to 8 h post-feeding (Fig. 5). Thus, these results indicate that the propionate pathway was considerably enriched in HR cows, whereas the acetate pathway was upregulated in LR cows.

## DISCUSSION

This study aimed to investigate whether microbiomes associated with rumen, bolus, and fecal samples differ between LR and HR cows and whether a distinct microbiome is associated with the HR phenotype. The overarching hypothesis of this study was that RT, ruminal microbiome, and individual VFA are linked, and low CH_4_ yield phenotype cows may have high RT, associated with distinct bacteria-archaeal cohorts, and a higher proportion of propionate compared to high CH_4_ yield phenotype cows. Hence, this is the first study reporting the link between distinct rumen microbiomes associated with distinct rumination phenotypes. Using the 16S rDNA bacterial diversity, metagenomic profiling, and VFA analysis, we were able to demonstrate that RT, microbiome, and VFA are linked, and RT can be used as a proxy for identifying efficient cows that naturally emit less CH_4_ than their peers. Additionally, methanogen communities may be conserved in bolus, rumen, and fecal microbiomes, indicating that bolus and fecal samples can serve as proxies for rumen samples. Therefore, this is also the first study to report the use of bolus and fecal samples as a proxy for rumen samples that can assist in identifying animals with low CH_4_-emitting potential.

Previously, we demonstrated the link between CH_4_ yield phenotype and the rumen microbiome in dairy cattle (17). Several studies have also reported that CH_4_ yield phenotypes are linked to different microbiomes in different ruminant species, such as sheep (26), beef cattle (25, 46), and our data in dairy cattle (17). In our previous study (17), we reported that a 22% difference in CH_4_ yield was associated with a 26% difference in gene copies of MCR (an enzyme that synthesizes CH_4_), indicating the strong connection between enzyme copy number and EME. Furthermore, low CH_4_ yield phenotype cows had lower acetate and higher propionate and, consequently, a lower acetate-to-propionate ratio compared to high CH_4_ yield phenotype cows. Although no differences in DMI and MY were noted in high and low CH_4_ yield cows, in Stepanchenko et al. (17), these cows were far along in their lactation cycle, and the difference in CH_4_ per DMI was comparable between Stepanchenko et al. (17) and the current study. These data reveal that CH_4_ yield microbiota are possibly adjusted by the host to maintain the phenotypic nature of the ruminant host. In low CH_4_ yield phenotypes, irrespective of the ruminant host, the methanogen community has a higher methanol-utilizing *Methanosphaera* profile in sheep (26) and dairy cattle (17), which also agrees with the findings of this study. The three species of *Methanosphaera* (ISO4, BMS, and *stadtmanae*) were found to be higher in HR cows not only in the rumen but also in bolus and fecal samples, indicating that methanogen communities are conserved in rumen, bolus, and fecal microbiomes. Furthermore, the turnover of H_2_ is much faster with less accumulation of H_2_, enabling *Methanosphaera* to survive, potentially outcompeting CO_2_-utilizing *Methanobrevibacter* populations. Notably, *Methanobrevibacter* YE315 appears to be a dominant member and was significantly lower in LR cows than HR cows, indicating that different species of *Methanobrevibacter* occur in the rumen of dairy cows and may contribute differently to total EME in LR and HR cows.

Previously, our study by Castaneda et al. (23) demonstrated that cows with a high RT phenotype were associated with lower EME and lower CH_4_ intensity (g CH_4_/kg milk) and greater CH_4_ efficiency (higher DMI intakes and higher MY in HR cows compared to LR). As the high RT phenotype has a similar microbial profile to that of CH_4_ yield phenotype, it may be inferred that high RT supports microbial niches that lead to less EME and higher productive efficiencies.

This study differed from Stepanchenko et al. (17) in the distribution of alternate H_2_-utilizing sinks. In Stepanchenko et al. (17), there were no significant differences in alternate H_2_ pathways, including any statistical differences in the gene copies of *mcrA* between high CH_4_ and low CH_4_ phenotype cows. This was surprising as we anticipated an increase in one or several alternate H_2_ pathways that increased in low CH_4_ cows when CH_4_ emissions were lower than in high CH_4_ cows. In contrast, in the current study, gene copies associated with sulfate reductase (*dsrAB*), fumarate reductase (*frdABCD*), nitrate reductase (*napA*), and ammonia-forming nitrite reductase (*nirS*) were all increased. This finding indicates that gene copies associated with alternate H_2_-utilizing pathways were increased in rumen samples from HR cows, thus supporting our hypothesis that higher RT supports higher turnover in the rumen and efficient distribution of H_2_ to alternate H_2_ sinks than methanogenesis. The most dominant sink also appears to be fumarate reductase across bolus, rumen, and fecal microbiomes, indicating better recycling of NADH and formation of succinate from fumarate (47, 48). It may also be possible that nitrogen is recycled better to accommodate ammonia formation and thus better support microbial synthesis (49). It was interesting to note that acetogenesis is enriched in fecal microbiomes, as one would expect that lower gut microbiomes are indeed enriched in acetogens (50). Although no differences between LR and HR cows were noted in acetogen populations in any of the sample types, further investigations using advanced approaches such as metatranscriptomics or quantification of acetyl CoA synthetase enzyme are required to determine whether acetogens proliferate under reduced methanogenesis conditions.

Interestingly, a consistent decrease in acetate and an increase in propionate in HR cows compared to LR cows indicates that HR cows have efficient microbial enzyme repertoires to support higher fermentation rates. Higher propionate levels were noted before the start of the study as well as towards the end of the study. Notably, these propionate levels were consistently higher throughout the 24 h period. An increase in bacteria, such as Unclassified *Clostridiales* 1, *Sharpea*, *Succiniclasticum*, Unclassified *Veillonellaceae*, and Unclassified *Succinivibrionaceae* in HR cows, is positively correlated with propionate formations. An enrichment of these bacteria accompanied by a higher increase in succinate and propionate has also been reported in low CH_4_ cows in Stepanchenko et al. (17) and Kaplan-Shabtai et al. (29). These data reveal that HR cows had similar microbial and VFA profiles as in the low CH_4_ phenotype in cows as described in Stepanchenko et al. (17), revealing that RT is a good proxy for CH_4_ yield phenotype, and RT may be a better marker to select cows for lower EME and better microbial efficiencies.

Finally, in the present study, we investigated the potential of bolus and fecal samples to serve as proxies for rumen microbial communities. In the 2 h post-feeding, we noted a significant reduction in Bacteroidetes accompanied by an increase in Firmicutes within the rumen microbial communities. Additionally, it was noteworthy that during the feeding process, there was a marked enhancement in microbial activity, which was evidenced by an increase in alpha diversity (species richness and diversity) and slightly distinct clustering patterns (weighted UniFrac) at 2 h post-feeding compared to other sampling hours. However, such fluctuations were not detected in the fecal communities, as these were not directly influenced by recent feed intake into the rumen. Despite minor variations in the relative abundance of the predominant bacterial populations, the microbial profiles and methanogen diversity exhibit consistency between bolus and rumen samples. However, it is imperative to exercise caution when collecting a bolus sample; this should occur while the cows are actively ruminating to ensure the collection of a representative sample from the regurgitated feed materials within the rumen. This sample may be obtained before feeding, 6 h post-feeding, or during active rumination. Fecal samples demonstrate an enrichment of Firmicutes, which is not surprising given the fiber-rich composition of fecal material. Although the Gram-negative bacterial populations in fecal samples are comparatively lower, the Firmicutes and the methanogen diversity appear to be similar in fecal, bolus, and rumen samples.

### Conclusions

This study highlights the potential of RT as a microbiome-linked, non-invasive strategy for identifying low EME cows. High-RT cows exhibited an increased abundance of genes associated with methylotrophic methanogenesis and alternative hydrogen sinks, alongside a greater enrichment of rapid-fermenting, propionate-producing bacteria. These microbial shifts suggest that host genotypes can shape rumination patterns to favor specific bacteria-methanogen interactions, enhancing rapid fermentation pathways while reducing hydrogen availability for methanogenesis. Furthermore, the observed diurnal fluctuations in bacterial communities, particularly the pronounced intraday variations in bolus microbiomes, reinforce the potential of bolus and fecal samples as proxies for rumen microbiomes in methane mitigation strategies. Leveraging RT as a selection criterion could provide a scalable and cost-effective approach to improving production efficiency and reducing methane emissions in dairy cattle. Additionally, integrating physiological traits like rumination behavior with bacterial-archaeal networks may offer new insights into genome-phenome interactions, enabling more efficient identification of methane-mitigating phenotypes.

## Data Availability

Both 16S rRNA and metagenomic raw sequence data have been submitted to the NCBI database under BioProject accession number PRJNA1215972.

## SUPPLEMENTAL MATERIAL

Data set S1 msphere.00090-25-s0001.xlsx Sequencing information for the samples analyzed in this study.

Data set S2 msphere.00090-25-s0002.xlsx Relative abundance of methanogenic and bacterial populations based on metagenomic analysis.

Data set S3 msphere.00090-25-s0003.xlsx Functional pathways linked to methanogenesis and alternative metabolic sinks.

Data set S4 msphere.00090-25-s0004.xlsx PERMANOVA of 16S rDNA bacterial communities in LR and HR Holstein cows.

Figure S1 msphere.00090-25-s0005.tif 16S rDNA bacterial diversity.

Legends msphere.00090-25-s0006.docx Legends for supplemental figure and tables.

ASM does not own the copyrights to Supplemental Material that may be linked to, or accessed through, an article. The authors have granted ASM a non-exclusive, world-wide license to publish the Supplemental Material files. Please contact the corresponding author directly for reuse.
