# Supplementary figures and images for: Host-specific microbiome-rumination interactions shape methane-yield phenotypes in dairy cattle
Source: mSphere. 2025 Apr 25;10(5):e00090-25. doi: 10.1128/msphere.00090-25 (PMC12108071; doi:10.1128/msphere.00090-25)

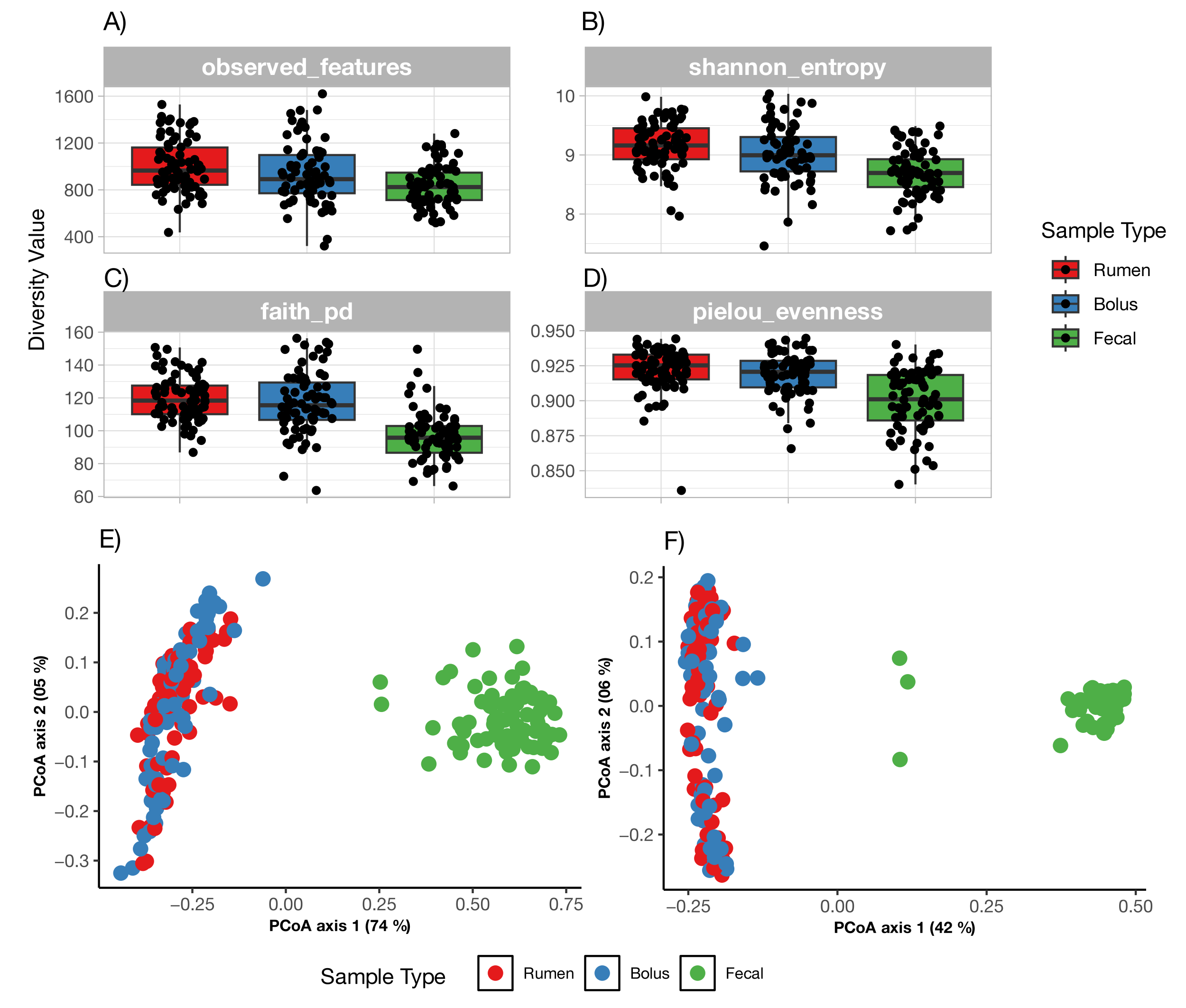

Supplement: Figure S1 — 16S rDNA bacterial diversity. [file msphere.00090-25-s0005.tif]
