# Supplementary material for: Host-specific microbiome-rumination interactions shape methane-yield phenotypes in dairy cattle
Source: mSphere. 2025 Apr 25;10(5):e00090-25. doi: 10.1128/msphere.00090-25 (PMC12108071; doi:10.1128/msphere.00090-25)
Supplement: Legends — for supplemental figure and tables. [file msphere.00090-25-s0006.docx]

**Supplementary dataset legends.**

**Dataset S1.** Sequencing information for the samples analyzed in this study. **A)** 16S rDNA sequencing details. **B)** Metagenomic sequencing details.

**Dataset S2.** Relative abundance (%) of methanogenic and bacterial populations based on metagenomic analysis. Data are presented for oral bolus (**A, B**), rumen (**C, D**), and fecal (**E, F**) samples from low (LR) and high (HR) ruminating Holstein cows at 2-hour and 8-hour sampling intervals. Values are expressed as mean ± SEM (Standard Error of the Mean).

**Dataset S3:** Functional pathways linked to methanogenesis and alternative metabolic sinks.
Data are shown for rumen (**A, B**), oral bolus (**C, D**), and fecal (**E, F**) samples from low (LR) and high (HR) ruminating Holstein cows at 2-hour and 8-hour sampling intervals. Values are expressed as mean ± SEM (Standard Error of the Mean).

**Dataset S4:** PERMANOVA (Permutational Multivariate Analysis of Variance) Analysis of 16S rDNA Bacterial Communities in low (LR) and high (HR) ruminating Holstein cows. Analyses were performed across different sampling hours (0,2,8 and 14) and sample types (Rumen, oral bolus, and fecal). Results are presented for A) Weighted UniFrac and B) Unweighted UniFrac. The **R² value** represents the proportion of variance explained by the model.

**Figure S1:** Assessment of rumen 16S rDNA bacterial diversity in ruminating Holstein cows across sample types (bolus, rumen, and feces). (A–D) Boxplots showing within-sample diversity (alpha diversity), measured by Pielou's evenness, Shannon diversity index, and Simpson diversity index. (E, F) Principal Coordinates Analysis (PCoA) plots based on weighted UniFrac (E) and unweighted UniFrac (F) distances, illustrating bacterial community composition stratified by sample type.
